# Maternal Diet and Selenium Concentration in Human Milk From an Italian Population

**DOI:** 10.2188/jea.JE20100183

**Published:** 2011-07-05

**Authors:** Francesca Valent, Milena Horvat, Darja Mazej, Vekoslava Stibilj, Fabio Barbone

**Affiliations:** 1 Institute of Hygiene and Clinical Epidemiology, University Hospital of Udine, Udine, Italy; 2 Jožef Stefan Institute, Ljubljana, Slovenia; 3 Institute of Hygiene and Epidemiology, Department of Medical and Biological Sciences, University of Udine, Udine, Italy

**Keywords:** selenium, human milk, maternal diet

## Abstract

**Background:**

Low selenium (Se) status is associated with several diseases. International organizations have proposed intakes of Se for general populations, including infants. Studies of the association of Se concentration in breast milk and maternal diet have yielded inconsistent results. We evaluated the relation between the intake of food items during pregnancy and Se concentration in human milk after delivery and compared infant intake of Se from breast milk with the recommended intakes.

**Methods:**

This cross-sectional study was part of the baseline assessment of a prospective cohort of Italian mother–child pairs enrolled in 1999–2001. Se concentration was measured in the milk of 100 women included in the cohort and correlated with the intake of food items during pregnancy and lactation as reported in a food frequency questionnaire.

**Results:**

Among foods consumed in pregnancy, only eggs had a positive, but weak, correlation with Se concentration in milk (*r* = 0.20, *P* = 0.04). Fish intake during lactation was also weakly correlated with Se in milk (*r* = 0.21, *P* = 0.04). Se content of breast milk in our population was lower than that noted in other international studies; however, very few children who were exclusively breastfed were estimated not to have met the recommended Se intake.

**Conclusions:**

Future research should aim to verify whether infants in this part of Italy still meet the recommended nutrient intake of Se and to assess the influence of the concurrent diet of lactating mothers on the Se content of their milk.

## INTRODUCTION

Selenium (Se) has a number of biological effects. In the human body, it has a role as an antioxidant, in thyroid hormone metabolism, in redox reactions, in reproduction, and in immune function. In humans, Se tissue levels are influenced by dietary intake, as Se is present in soil and enters the food chain through plants. Se deficiency is rare and causes 2 diseases, both of which occur in areas of China where the soil is deficient in Se: Keshan disease (an endemic cardiomyopathy that affects children, adolescents, and young women) and Kashin-Beck disease (an endemic osteoarthritis also known as “Big Bone Disease” that leads to stunted growth and deformity of the joints).^1^ In addition, low Se status has been associated with several other chronic diseases, such as cancer, cardiovascular disease, and asthma.^2^

Estimates of Se requirements have been based not only on the epidemiologic evidence from the areas of China with endemic or nonendemic Keshan disease, but also on the attempt to maximize the enzymatic activity of glutathione peroxidases (GPx), the selenoproteins through which Se exerts its biological effects.^3^ Se requirements for the prevention of chronic diseases such as cancer may be even higher.^3^ Although various countries have used the criterion of maximization of plasma GPx, different Se intakes are recommended around the world.^3^ A Joint Food and Agriculture Organization/World Health Organization (FAO/WHO) Expert Committee on Human Vitamin and Mineral Requirements proposed a recommended nutrient intake (RNI) for Se of 6 µg/day in infants aged 0 to 6 months weighing approximately 6 kg.^4^ However, no functional indicator of Se status has been demonstrated that reflects response to dietary intake in infants. In addition, there are no reports of full-term US or Canadian infants who were exclusively and freely fed human milk and showed signs of selenium deficiency. Therefore, the US Institute of Medicine has based its recommendations on an adequate intake (AI) that reflects the observed mean selenium intake of infants fed principally with human milk, ie, 15 µg/day.^5^ The same organization set a tolerable upper intake (UI) level of 45 µg/day for infants aged 0 to 6 months.^6^

The recommended levels are generally assumed to be met by breast-fed infants, given their intake of milk (approximately 0.8 L/day) and the range of Se content of breast milk that has been reported in many parts of the world.^4^^,^^5^

Se in human milk was reported to vary with maternal Se intake in a Finnish study,^7^ although in a recent small study conducted in Texas among low-income lactating women of Mexican-American heritage no correlation was observed between dietary Se estimated through 24-hour recall and breast milk concentration.^8^

To evaluate the relation between dietary habits during pregnancy and Se concentration in human milk after delivery and to compare infant intake of Se from breast milk with recommended intakes, we estimated Se content in the breast milk of a sample of Italian mothers.

## METHODS

### Study subjects

This cross-sectional study was part of the baseline assessment of a cohort of mothers living in 17 towns of the Friuli Venezia Giulia region, Northeastern Italy, who delivered a baby from 1 April 1999 to 31 May 2001. The main objective of the cohort study was to assess the association of intrauterine exposure to low mercury concentrations after maternal fish consumption during pregnancy with future child neurodevelopment. A detailed description of the rationale and procedures of the cohort enrolment is reported elsewhere.^9^ In brief, two thirds of the women contacted agreed to participate in the study, and the overall cohort included 242 women. All the women were informed about the study and provided written consent to take part in the research.

### Dietary assessment

A semi-structured questionnaire was administered by a trained interviewer in their homes, approximately 3 months after delivery. The questionnaire collected sociodemographic information and residential and occupational history of the parents, pregnancy history, and maternal frequency of consumption of 47 food items during pregnancy and of 3 fish items (fresh fish, seafood, and canned fish) during lactation. The food frequency questions were adapted from a previously validated food frequency questionnaire.^10^^–^^12^

### Collection of breast milk samples

At the time of the interview, a sample of 10 mL of breast milk adequate for biochemical analysis was collected among lactating women. To maximize participation and minimize discomfort, the women were allowed to collect their breast milk at any time they felt comfortable and no indication was provided to them on when to express it with respect to the infant’s feeding. The sample of milk was stored at −20°C until the chemical analysis was performed.

### Chemical analysis of Se in breast milk

For the analysis of Se,^13^ 1 g of milk sample that had been previously heated to 38°C and shaken was weighed into a Teflon tube (50 mL, Savillex). One mL each of 96% H_2_SO_4_ and 65% HNO_3_ were added, and the tube was capped and then heated in an aluminium block maintained at 125°C for 30 min. After cooling, 5 additions (3 × 1 mL, 2 × 2 mL) of 30% H_2_O_2_ were made, and the tube was reheated for 10 min at 125°C after every addition. The solution was then cooled to room temperature. To reduce selenium to Se^4+^, 2.5 mL of concentrated HCl was added to the solution, which was heated for 10 min at 100°C. Finally, the solution was diluted to 10 g with MilliQ water, and selenium was measured by the hydride generation-atomic fluorescence spectrometry (HG-AFS) system. In the same way and with the same amounts of chemicals as for samples, a process blank was run every time. Each sample was analyzed twice in 1 day. Standard, sample, and blank solutions were measured at least twice. The limit of detection (LOD) of the methods was 2.5 ng/mL, estimated on the basis of 3 standard deviations of the blank, and expanded uncertainty (coverage factor was 2) was 10%.^13^

### Statistical analysis

The distribution of Se concentration in breast milk was described by the mean, standard deviation (SD), quartiles, minimum, and maximum values. The normality of Se distribution was assessed using the Shapiro-Wilk test. Because the distribution was not normal, the difference between women who were exclusively breastfeeding and those who were not was tested using the Wilcoxon rank sum test. Spearman correlations were calculated to assess the associations between Se concentration in breast milk and the reported weekly consumption of each food item. Individual items were then grouped to form broader food categories (vegetables; fruit; milk and dairy products; meat; all fish and seafood; pasta, rice, bread, pizza, cakes) by summing the estimated intakes (servings/week) of each food item in a certain category, and the correlation between their frequency of consumption with the Se content of milk was also tested. *P* values less than 0.05 were considered statistically significant.

The adequacy of infant Se intake from breast milk was assessed among exclusively breastfed infants only. The estimated daily Se intake was calculated by multiplying milk intake (assuming a daily intake of 800 mL of breast milk) by the Se content in their mothers’ milk.

## RESULTS

A total of 139 mothers (57%) were still exclusively breastfeeding their children at the time of the interview, whereas the remaining women integrated their infant feeding with variable proportions of formula. An adequate sample of breast milk was provided by 100 women, among whom 82 were exclusively breastfeeding. Mean age at delivery of mothers who provided the sample of breast milk was 30.0 ± 4.7 years (median 30, range 18–42). Twenty-seven had a middle school diploma, 60 had a high school diploma, and 13 had a university degree.

**Figure 1.**
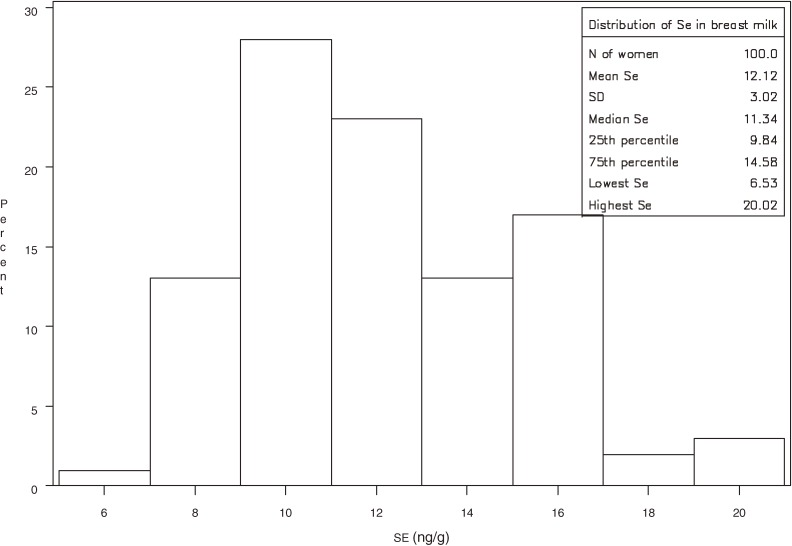
Distribution of Se concentration* (ng/g) in breast milk of 100 Italian lactating women. *As determined by hydride generation-atomic fluorescence spectrometry (HG-AFS)

The mean Se concentration in milk samples was 12.1 ± 3.0 ng/g (25th percentile 9.8, median 11.3, 75th percentile 14.6, range 6.5–20.0; Figure 1). The mean Se content of milk was slightly higher among women who were not exclusively breastfeeding (12.9 ± 3.0 ng/g, median 11.8) than among those who were (11.9 ± 3.0 ng/g, median 11.3), but the difference was not statistically significant (*P* = 0.22).

Among the foods consumed during pregnancy, only eggs were positively correlated with breast milk Se (*r* = 0.20, *P* = 0.04; Table 1). Marginally significant inverse associations were found for consumption of fruit juice (*r* = −0.20, *P* = 0.05), cheese (*r* = −0.19, *P* = 0.06), and apples and pears (*r* = −0.18, *P* = 0.07). Fresh fish and seafood intakes in pregnancy were weakly associated with Se content of maternal milk (*r* = 0.12, *P* = 0.21 and *r* = 0.15, *P* = 0.14, respectively). No significant correlation was observed when food items were grouped into broader categories, except for a marginally significant inverse correlation with fruit consumption (Table 1). In addition, no significant association was observed between intake of multivitamin supplements during pregnancy and Se in breast milk (mean 10.9 ± 3.3 ng/g, median 9.6, and mean 12.3 ± 2.9 ng/g, median 11.4, respectively, among women who did and did not consume multivitamin supplements, *P* = 0.11).

**Table 1.** Correlation between reported weekly intake of food items during pregnancy and lactation and Se concentration in breast milk 3 months after delivery among 100 Italian women

Food item (serving size) in pregnancy	Spearman correlation coefficient *r* (*P*-value)
Milk (1 glass)	−0.09 (0.41)
Yoghurt (1, 125 g)	−0.05 (0.62)
Pasta or rice with oil or butter (80 g)	−0.10 (0.31)
Pasta or rice with tomato sauce (80 g)	0.05 (0.58)
Pasta or rice with meat sauce (80 g)	−0.03 (0.79)
Pasta or rice with fish sauce (80 g)	0.06 (0.55)
Soup with vegetables or legumes (250 g)	0.11 (0.29)
Fish soup (100 g)	0.10 (0.34)
Pizza (1 slice, 200 g)	−0.05 (0.61)
Bread (1 piece/slice)	−0.04 (0.70)
Crackers, bread sticks, crispy bread (1 pack/5/3)	0.06 (0.58)
Polenta (2 slices)	0.03 (0.76)
Boiled or grilled chicken or turkey (200 g)	−0.13 (0.18)
Roasted, stewed, or fried chicken or turkey (200 g)	0.03 (0.75)
Boiled or grilled beef or pork (120 g)	0.04 (0.71)
Roasted, stewed, or fried beef or pork (150 g)	0.00 (0.99)
Mixed fried fish (150 g)	0.03 (0.74)
Mixed grilled fish (150 g)	0.11 (0.27)
Ham (50 g)	−0.12 (0.21)
Eggs (1)	0.20 (0.04)
Cheese (100 g)	−0.19 (0.06)
Cakes (1 slice)	−0.09 (0.40)
Potatoes (1, 150 g)	−0.01 (0.89)
Mixed salad (tomatoes, cucumbers, carrots) (50 g)	−0.05 (0.62)
Green salad and radicchio (50 g)	0.00 (0.97)
Spinach (200 g)	0.09 (0.38)
Cabbage, cauliflower, broccoli, ​ Brussels sprouts, Turnip greens (125 g)	−0.07 (0.50)
Carrots (100 g)	−0.00 (0.97)
Tomatoes (150 g)	−0.05 (0.64)
Peppers, zucchini, eggplant (150 g)	0.09 (0.38)
Artichokes (1, 3 bottoms)	−0.09 (0.36)
Fennel (1100 g)	−0.02 (0.83)
Legumes (green peas, beans, chickpeas, ​ green beans, lentils, etc.) (100 g)	−0.07 (0.49)
Bananas (1)	−0.09 (0.36)
Apples or pears (1)	−0.18 (0.07)
Peaches (1)/apricots (3)/plums (3), (100 g)	−0.14 (0.18)
Melon (2 slices)/watermelon (1 slice)	−0.11 (0.25)
Strawberries/cherries (1 cup, 150 g)	0.04 (0.71)
Oranges (1)/grapefruits (1)/lemons (1) ​ mandarins (2)/citrus fruit juices (150 g)	−0.04 (0.69)
Grapes	−0.05 (0.60)
Kiwi	0.04 (0.69)
Fruit juice (1 small bottle, 125 g)	−0.20 (0.05)
Dried fruit (50 g)	−0.02 (0.86)
Cooked fruit (1 cup, 150 g)	0.08 (0.44)

Summary questions on fish during pregnancy	
Fresh fish (150 g)	0.12 (0.21)
Seafood (150 g)	0.15 (0.14)
Canned fish (1 can, 80 g)	0.00 (0.98)

Food groups (servings) during pregnancy	
Vegetables	−0.02 (0.80)
Fruit	−0.18 (0.07)
Milk and dairy products	−0.16 (0.12)
Meat	−0.06 (0.54)
All fish and seafood	0.12 (0.22)
Pasta, rice, bread, pizza, cakes	−0.01 (0.89)

Summary questions on fish during lactation	
Fresh fish (150 g)	0.21 (0.04)
Seafood (150 g)	0.16 (0.10)
Canned fish (1 can, 80 g)	0.03 (0.76)

Regarding the consumption of selected seafood items during lactation, Se concentration in mother’s milk was significantly correlated with current fresh fish consumption (*r* = 0.21, *P* = 0.04) and marginally correlated with current intake of seafood (*r* = 0.16, *P* = 0.10). There was no association with current intake of canned fish (*r* = 0.03, *P* = 0.76).

**Figure 2.**
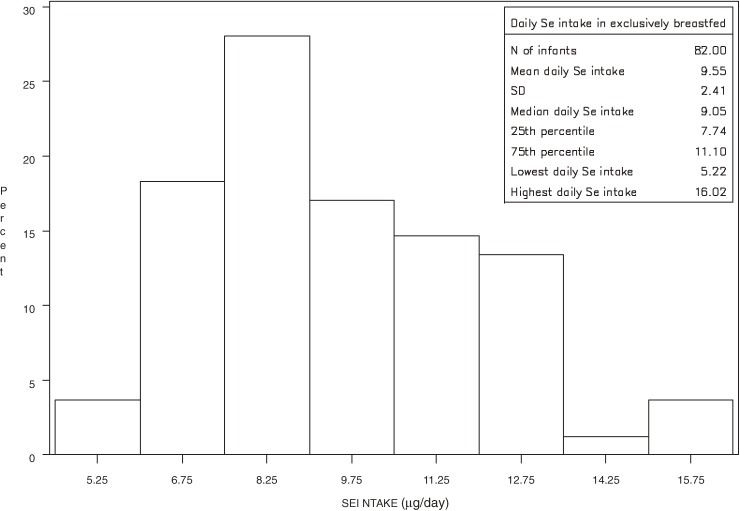
Distribution of daily Se intake* (µg) among 82 exclusively breastfed infants in Italy. *Estimated for each exclusively breastfed infant by multiplying milk intake (assuming a daily intake of 800 mL of breast milk) by the Se content in his/her mother’s milk

Among the 82 exclusively breastfed infants, the estimated mean daily Se intake was 9.5 ± 2.4 µg; the minimum intake was 5.2 µg, and the maximum intake was 16.0 µg (Figure 2). Four children had a daily Se intake lower than 6 µg (5.2 µg in 1 child and 5.8 µg in 3 children).

## DISCUSSION

In our population, Se concentration in breast milk at approximately 3 months after delivery was similar to that in other European populations, but generally lower than that observed in many other studies worldwide of relatively mature milk (Table 2).^8^^,^^14^^–^^25^

**Table 2.** Comparison of Se levels in mature human milk in different geographical areas

Country	Se concentration (ng/mL)	Measure	*n*	Reference	Estimated infant Se intake (µg/day)^a^
Italy	12.1, 3.0^b^	Mean, SD	100	Present study	9.7 (a)^c^
	11.3^b^	Median			9.5 (b)^c^
Finland	10.9, 1.9	Mean, SD	18	Kumpulainen et al, 1984^11^	8.7
Finland	16.4, 3.2 (a)^d^	Mean, SD	175	Kantola and Vartiainen, 2001^12^	13.2
	18.9, 3.0 (b)^d^		81		15.1
Gambia	15.3, 1.2 (a)^e^	Mean, SEM	8	Funk et al, 1990^13^	12.2
	21.0, 0.9 (b)^e^		15		16.8
Greece	18, 3	Mean, SD	8	Bratakos and Ioannou, 1991^14^	14.4
Guatemala	19.2	Median	84	WHO, 1989^15^	15.4
Hungary	13.9	Median	71	WHO, 1989^15^	11.1
Japan	17	Median	10	Higashi, 1983^16^	13.6
Japan	15, 6	Mean, SD	134	Yamawaki et al, 2005^17^	12
Nepal	10.0, 1.0	Mean, SEM	26	Moser et al, 1988^18^	8
Philippines	33.2	Median	65	WHO, 1989^15^	26.6
Poland	10.51, 2.76	Mean, SD	352	Zachara and Pilecki, 2001^19^	8.4
Spain	16.3, 4.7	Mean, SD	31	Navarro-Blasco and Alvarez-Galindo, 2004^20^	13.4
Sweden	13.1	Median	32	WHO, 1989^15^	10.5
USA	15.1, 5.8	Mean, SD	8	Smith et al, 1982^21^	12.1
USA	15, 1	Mean, SD	10	Levander et al, 1987^22^	12
USA	15.7, 5.3	Mean, SD	10	Hannan et al, 2009^8^	12.6
Zaire	19.3	Median	69	WHO, 1989^15^	15.4

Abbreviations: SD, standard deviation; SEM, standard error of the mean.

^a^Estimated by the authors, assuming that the intake of breast milk was 800 mL/day for all children in the studies reported in the table and using either the mean or the median Se content of breast milk, depending on data availability.

^b^ng/g.

^c^(a) All children, assuming that the daily intake of breast milk was 800 mL/day; (b) Exclusively breastfed children.

^d^(a) Before addition of sodium selenate to all agricultural fertilizers; (b) After addition of sodium selenate to all agricultural fertilizers.

^e^(a) Rainy season; (b) Dry season.

Among almost 50 food items consumed in pregnancy, only egg consumption was significantly positively correlated with Se concentration in maternal milk. Inverse relations were found with cheese, fruit juice, and fruit consumption in pregnancy. Because we considered a large number of items consumed by women in pregnancy, we cannot exclude the possibility that the observed associations were due to chance alone. However, when we considered the frequency of current fish consumption (ie, at the time of milk collection), we observed a moderately significant positive correlation of fish intake with Se concentration in breast milk and a borderline significant correlation with other seafood. In 1987, a study by Schubert et al identified Se core foods based on their Se concentration as reported in analytical studies published since 1960 and their frequency of consumption in the United States as estimated by the Nationwide Food Consumption Survey in 1977–78 by the US Department of Agriculture. Of 114 food items, the 5 most highly ranked food aggregates were beef, white bread, pork, chicken, and eggs, which provided half of the Se accounted for in the diets of the survey respondents.^26^ Nutrient daily values (DVs) are reference numbers developed by the US Food and Drug Administration to help consumers determine if a food contains a large or small amount of a specific nutrient; percent DV indicates the percentage of the DV provided in 1 serving: 5% is considered low, 10% to 19% is a source, and 20% or more is high. According to the US National Institutes of Health Office of Dietary Supplements, food items high in Se (ie, providing ≥20% of the nutrient DV) include Brazil nuts (544 µg/ounce, 780% of the DV), light tuna canned in oil (drained, 63 µg/3 ounces, 95% DV), beef, turkey, and chicken (20–35 µg/3–3.5 ounces), spaghetti, noodles, and macaroni (15–34 µg/serving), cod (32 µg/3 ounces), and eggs (14 µg/1 medium egg).^27^ The Se content of foods, however, can be extremely variable, depending on the combination of geologic/environmental factors and Se supplementation of fertilizers and animal feed stuffs. In fact, according to the Italian Food Composition Database for Epidemiological Studies in Italy^28^ the food items that are the richest in Se are several species of fish and other marine foods (approximately 1.5–6 times higher in Se than meats), which is consistent with the positive correlation of Se in breast milk with concurrent fish and seafood intake. In our population, however, it could be that the combination of food intakes and Se content in food resulted in the absence of observed associations for particular food groups consumed during pregnancy, thus explaining the paucity of associations of Se concentration in milk with food intakes at that time.

Se bioavailability is also an important factor that could explain the scarcity of associations between dietary intakes in pregnancy and Se concentration in mature milk. In fact, there may be large variations in the Se content of foods and in the chemical forms of the element that are absorbed and metabolized.^29^ For example, the retention of organic forms is higher than that of inorganic forms and some functional biomarkers respond differently to the various species.^30^ Overall, absorption of all forms of Se is relatively high (70%–95%), but varies according to the source and the Se status of the subject. Wheat and meat (excluding fish) are considered the most important dietary sources of Se. On the other hand, although the Se content of fish is relatively high, its bioavailability is reported to be low.^31^ The varying bioavailability of Se from different sources was also noted in a study by Finley et al,^32^ who examined utilization of ^75^Se in broccoli and meat by feeding rats a diet with adequate or high levels of Se. When dietary Se was adequate, more ^75^Se from pork than from broccoli was retained in tissues, whereas no significant differences were observed when dietary Se was high.

Another important factor is the method of assessing Se status in humans. To cite an example, a systematic review showed that plasma, erythrocyte, and whole-blood Se, plasma selenoprotein *P*, and plasma, platelet, and whole-blood glutathione peroxidase activity responded to changes in Se intake in clinical trials; however, evidence of the usefulness of other potential biomarkers of Se status was insufficient. In addition, there is great heterogeneity between studies, suggesting that there may be circumstances under which particular biomarkers have different utility (eg, baseline Se level, genotype, intake level, and duration).^33^ Under some circumstances, therefore, Se in breast milk may not reflect Se status. In fact, in a study by Hannan et al no significant correlation was found between Se dietary intake as estimated from a 24-hour recall and Se in milk.^8^ Another study, by Debski et al, found that Se concentration in breast milk was significantly higher among lacto-ovo-vegetarian women than among nonvegetarian women, despite similar concentrations of protein in milk.^34^ When the authors calculated mean dietary Se intakes, however, they were similar in vegetarian and nonvegetarian mothers,^34^ which is consistent with our finding that the Se content of milk may not be strongly correlated with dietary intake of Se.

In the present study, we assessed the association between the usual frequency of food consumption in pregnancy and the Se content of breast milk approximately 3 months after delivery. Se has 4 different plasma forms, with a biological half-life of 0.2 to 285 hours, and persists in the tissue pool for 115 to 285 days.^35^ The kinetics of Se likely explain the lack of association with intakes of some foods, including fish, consumed several months before. In fact, when we assessed current (ie, during lactation) fish consumption, correlations were observed between fresh fish and seafood and Se in breast milk.

Because diet was assessed retrospectively in this study, recall bias could be an issue; however, it is unlikely that it affected the results. In fact, the interview mode (face-to-face interviews with a trained interviewer) and the short time elapsed between the end of pregnancy and the interview should have minimized such bias.

In addition to maternal diet, other sources of variability in the Se content of breast milk have been reported. First, there is wide interindividual variation in the Se content of human milk.^19^ In addition, the Se content of colostrum is higher than that of transitional and mature milk.^19^^,^^24^^,^^36^ Higashi et al found that Se content declined significantly during the first month of lactation and then reached a plateau; thus, this source of variability should not be an important issue because all milk samples were collected at approximately the same infant age, approximately 3 months.^19^ Finally, the Se content of hindmilk (milk at the end of an infant feeding) is greater than that of foremilk (milk at the beginning of the feeding).^24^ In particular, Smith et al found that the Se concentration of foremilk and hindmilk from 5 lactating women was 15.9 ± 3.4 and 14.1 ± 3.2 ng/mL, respectively, at 2 months and 16.4 ± 3.1 and 13.9 ± 3.2 ng/mL at 3 months.^24^ This could explain part of the variability observed in our population; in fact, the women were allowed to collect their breast milk at any time they felt comfortable, and no indication was provided to them on when to express it with respect to infant feeding. Data collected by Smith at al^24^ suggest that variability in Se concentration in our study might have been as high as 2 ng/mL, due to the phase of nursing when women expressed milk. In contrast, there is no evidence that time of day influences the Se content of milk.^24^

Despite the large variability in Se concentration in the breast milk of our study population, almost all infants who were exclusively breastfed were estimated to meet the RNI established by WHO/FAO in 1989 for that age group^4^: in only 4 cases was the intake between 5 and 6 µg/day. Almost all children in our study group, however, had intakes lower than the observed mean Se intake of infants fed principally with human milk in the United States and Canada, ie, 15 µg/day, which is considered an AI in those countries.^5^

The Se content of breast milk in this cohort may not be representative of the regional population; in fact, not all the women who were contacted agreed to participate in the study, and not all the women who formed the cohort could or wanted to provide a sample of breast milk. However, there is no obvious reason for women who participated in the study to have Se concentrations in milk that systematically differ from those of nonparticipants.

In addition, Se concentrations in milk could have changed with time due to possible alterations in the dietary habits in the population, chemical composition of fertilizers and animal feed stuffs, or geographic origin of grains, fish, and other foods. Therefore, periodic assessment of Se concentration in breast milk among the regional population is warranted so as to exclude inadequate Se intake of some breastfed infants. The collection of multiple milk samples at various child ages and at different feeding phases (ie, foremilk and hindmilk) would aid in more precisely determining actual infant intake of Se from maternal milk.

In conclusion, this study showed that Se concentration in the breast milk of a sample of Italian lactating mothers and Se intake among their infants met the recommendations established by WHO/FAO^4^ in more than 95% of cases; however, on average, intakes were lower than in other countries, as shown in Table 2. Se content of breast milk 3 months after delivery was poorly correlated with reported intakes of food during pregnancy and weakly correlated with concurrent fish intake. We cannot ignore this association of Se in breast milk with concurrent fish consumption when providing nutrition recommendations to lactating mothers; indeed, dietary guidelines should take into account both the potential risks (eg, mercury) and benefits (eg, Se, polyunsaturated fatty acids) of eating fish. Future research should aim to verify whether infants in this part of Italy still meet the RNI of Se and to assess the influence of concurrent diet of lactating mothers on Se content of their milk.
